# Data-Driven Personalization of a Physiotherapy Care Pathway: Case Study of Posture Scanning

**DOI:** 10.2196/18508

**Published:** 2020-09-15

**Authors:** Olli Korhonen, Karin Väyrynen, Tino Krautwald, Glenn Bilby, Hedwig Anna Theresia Broers, Guido Giunti, Minna Isomursu

**Affiliations:** 1 University of Oulu Oulu Finland; 2 Qinematic Stockholm Sweden; 3 Blekinge Institute of Technology Karlshamn Sweden; 4 Bright Cape Eindhoven Netherlands

**Keywords:** digital health services, information systems, case reports, qualitative research, physiotherapy, posture

## Abstract

**Background:**

Advanced sensor, measurement, and analytics technologies are enabling entirely new ways to deliver health care. The increased availability of digital data can be used for data-driven personalization of care. Data-driven personalization can complement expert-driven personalization by providing support for decision making or even by automating some parts of decision making in relation to the care process.

**Objective:**

The aim of this study was to analyze how digital data acquired from posture scanning can enhance physiotherapy services and enable more personalized delivery of physiotherapy.

**Methods:**

A case study was conducted with a company that designed a posture scan recording system (PSRS), which is an information system that can digitally record, measure, and report human movement for use in physiotherapy. Data were collected through interviews with different stakeholders, such as health care professionals, health care users, and the information system provider, and were analyzed thematically.

**Results:**

Based on the results of our thematic analysis, we propose three different types of support that posture scanning data can provide to enhance and enable more personalized delivery of physiotherapy: 1) modeling the condition, in which the posture scanning data are used to detect and understand the health care user’s condition and the root cause of the possible pain; 2) visualization for shared understanding, in which the posture scanning data are used to provide information to the health care user and involve them in more collaborative decision-making regarding their care; and 3) evaluating the impact of the intervention, in which the posture scanning data are used to evaluate the care progress and impact of the intervention.

**Conclusions:**

The adoption of digital tools in physiotherapy has remained low. Physiotherapy has also lacked digital tools and means to inform and involve the health care user in their care in a person-centered manner. In this study, we gathered insights from different stakeholders to provide understanding of how the availability of digital posture scanning data can enhance and enable personalized physiotherapy services.

## Introduction

### Background

Health care is becoming increasingly data-driven [1,2]. Availability of digital data creates opportunities to deliver care in entirely new ways and to enhance current care models. Collectively, data-driven health care is enabled by information systems that seamlessly integrate the data and best care practices for health service delivery [3,4]. Information systems are powerful tools that can capture, store, process, and communicate timely information that can be used for the personalization of care [5] and can provide the right information to the right person in the right format [6]. Big data approaches [7], telehealth technologies [8], novel interactive visualization techniques [9], and artificial intelligence (AI)–based solutions [10] represent some ways in which information systems can be used to enable care that is more data-driven but is also aligned with the needs of the individual health care user in a personalized manner. Current care models highlight the importance of placing the health care user at the center of their care [11], which is often referred to as patient-centered care [12]. Personalization can be used to empower health care users to understand what matters to them [13].

Researchers have envisioned that in this form of participatory health care, the role of the health care professional is to complement the health care user’s own resources in managing their health so that the combined resources of these stakeholders will lead to the most optimal decisions regarding care [6]. A participatory approach to health care is often applied in physiotherapy, where user involvement is imperative for care, as the health care user drives the intervention and participates in decisions on how the intervention is sustained [14,15].

In health care, personalization often takes place in the interactions between the stakeholders; it involves the collective use of different health care technologies and the health care service system where the service is delivered [16,17]. However, in the field of personalization, there is a lack of research focusing on personalization at the level of the entire care process [17] and on the use of theoretical personalization frameworks in the design of health care technologies [18]. Increasing numbers of health care technologies are becoming available for different chronic conditions [19]; however, in physiotherapy, a common problem is that health care users are not readily provided with tools and information that enable them to participate in the care process in a person-centered manner [20]. 

Many studies have concluded that adoption of digital tools to support rehabilitation practice remains low, even though physiotherapists see potential in digitally supported rehabilitation [21]. Personalization has been found to be a key factor in acceptability and adoption of digital technologies in a rehabilitation context and in value creation [22,23]. Previous attempts to achieve more personalized physiotherapy with adoption of information systems include a checklist for choosing the most suitable health care users for blended physiotherapy [24] and an investigation of how adoption of information systems alters bodily action during clinical encounters and perception of agency in care-related interactions [25].

Service design is a set of design methods which integrate the possibilities and means to design and deliver a service while keeping the stakeholders, context, and other service development challenges at its heart [26]. Service design takes all the different stakeholders who are part of the service delivery into consideration to understand their needs and expectations [27,28]; this also applies to health care [29]. The importance of considering the health care user’s experience and the service encounters between the health care user and health care provider in health care service design has been identified in prior research [30]. However, in the design of health care technologies, the viewpoints of relevant stakeholders who are involved with and impacted by the service system are still often not addressed [31].

### Goals

In this study, we aimed to investigate how the availability of a new set of digital data, namely posture scanning data, can enhance and enable more personalized delivery of physiotherapy. The research question we asked is: *How does posture scanning data enable more personalized delivery of physiotherapy?*

## Methods

### Study Design

To answer our research question, we conducted a case study. Case studies are useful when there is a need to understand a complex social phenomenon that occurs in a real-world setting [32,33]. Our unit of analysis was an information system called a posture scan recording system (PSRS). The PSRS can be used to digitally record, measure, and report human movement when a health care user visits a physiotherapist. We selected this specific case because it allowed us to explore a real-world setting where an information system is used in a health care context for personalizing care to fit the needs of a specific health care user. Our aim was to gain insights from the different stakeholders involved in physiotherapy: the developers of the PSRS as well as the health care professionals and health care users who can use the PSRS to design novel personalized health care services in physiotherapy.

### Setting

The study was conducted as part of a development and research project involving four partners in three countries: Finland, Sweden, and the Netherlands. The PSRS was designed by the Swedish company Qinematic AB, has been commercially available since 2017, and has been continuously developed since then. The data collection and analysis were led by the University of Oulu in Finland in collaboration with the Swedish research institute RISE SICS and Bright Cape. Interviews with Qinematic personnel and the health care users were conducted in Sweden. Interviews with the health care professionals were conducted in the Netherlands.

### The PSRS

The PSRS is an information system solution that can be used in physiotherapy to record, measure, analyze, and report a health care user’s movement and posture at the point of care. This functionality is expected to increase the personalization of care by providing a more accurate understanding of the physiology of a specific health care user, better communication of the user’s medical condition and the effectiveness of care, and better targeting of care. The PSRS consists of two main information system components: Qinematic Posture Scan and Qinematic Movement Lab. The first component, PSRS Posture Scan, is used by the health care professional to objectively assess the health care user’s movement through an image processing system. The second component, the PSRS Movement Lab, is used as a communication and reporting tool between the health care professional and the health care user. Health care professionals can use this system to create a therapy training plan that includes rehabilitation-related exercises (type, interval, and frequency of exercise), administer questionnaires, and schedule follow-up physiotherapy assessments. Health care users can report their exercise adherence and respond to prompted questionnaires (eg, to report pain).

At the time of the interviews, the PSRS Posture Scan was fully developed and in use, whereas the PSRS Movement Lab was still under development. Therefore, as no functional software for the PSRS Movement Lab existed at the time, a paper prototype [34] of the PSRS Movement Lab was used to illustrate its implemented and future functionalities to the health care users and professionals. A detailed description of the PSRS in the form of a storyboard is provided for more context.

### PSRS Storyboard

**Figure 1:**
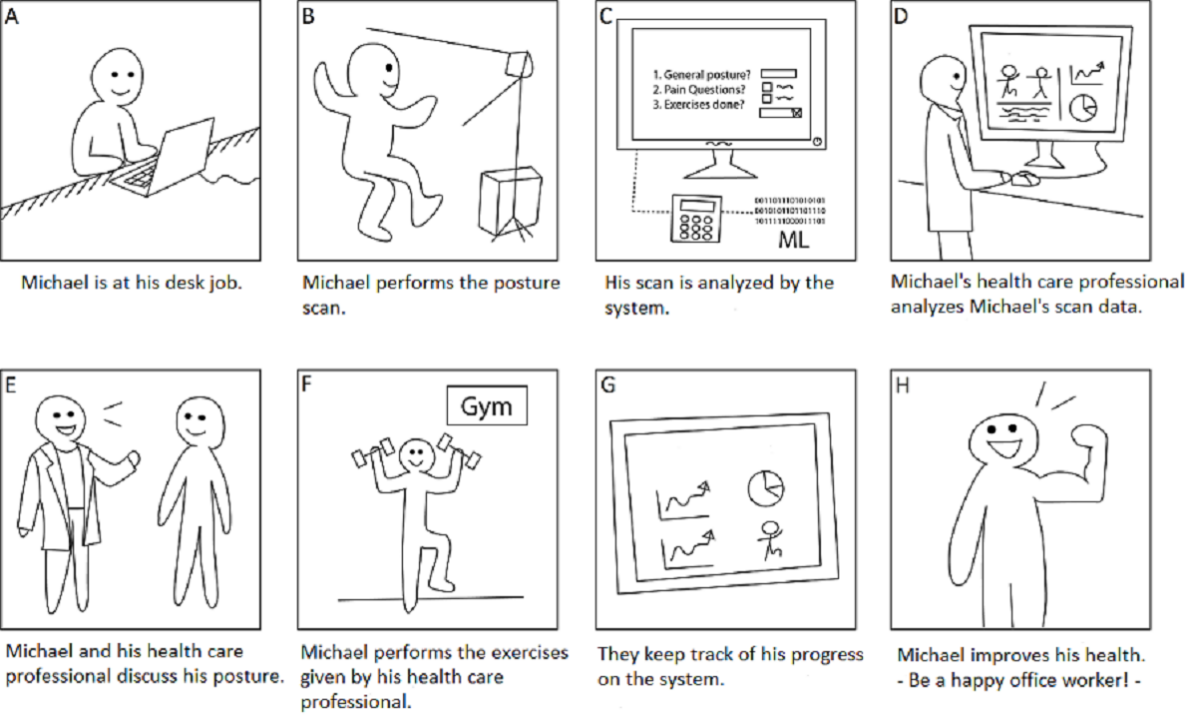
Storyboard depicting the use of posture scanning in the physiotherapy care pathway.

A storyboard is a narration that describes the stakeholders’ activities when using a service [35]. We used a storyboard to illustrate the roles of the different stakeholders and the posture scanning in the physiotherapy care pathway using a fictional persona, a health care user named Michael. Care pathways (also known as clinical pathways) are longitudinal and multidisciplinary treatment plans that describe all desired diagnostic and treatment steps for ensuring coordination and continuity of care [36,37]. The storyboard of the physiotherapy care pathway is depicted in Figure 1.

The storyboard starts by presenting Michael, a sedentary worker who suffers from pain and decreased movement (A). Michael consults a health care professional; after providing certain information, such as gender, age, body weight, a discomfort report with International Classification of Disease, Tenth Revision (ICD-10) comfort pain areas, and a fall report as a potential indicator of inability to perform the movements to be scanned, he is scanned by the Posture Scan at the point of care (B). The Posture Scan uses machine learning algorithms to analyze Michael’s movement levels (C). Once the scan is completed successfully, a report about Michael’s movement is generated for Michael and the health care professional. The health care professional analyzes the aggregated data (D) and discusses the results with Michael (E) to create a personal training plan for Michael using Movement Lab. Michael can see the therapy training using the Movement Lab application and commits to performing the prescribed exercises at home (F). Michael reports his progress to the health care professional using Movement Lab (G). After some time, Michael’s posture may improve (H), and the improvement will become visible in a new Posture Scan. Between the initial posture scan and the possible improvement of Michael’s posture, Michael and the therapist may be in contact via the PSRS Movement Lab and may have physical meetings to evaluate the effects of the exercises and adjust them if necessary.

### Data Collection

The interviews represent our primary data. The data were collected between February and May 2018. The participants were informed of the nature of the study and signed an informed consent form. The stakeholders interviewed were Qinematic staff, healthy individuals who represented health care users, and health care professionals in the physical therapy domain. In the interviews, we followed a protocol where one researcher always led the conversation while a second researcher took field notes. All interviews were audio-recorded and transcribed. Interviews with Qinematic staff and health care users were conducted in English, while the interviews with health care professionals were conducted in Dutch. Dutch to English translation was performed by AB, who conducted the interviews with the health care professionals and is a native Dutch speaker.

1. Qinematic staff: We conducted a group interview (90 minutes) with a service designer and a human movement scientist. The aim was to gain a general understanding of the design and development of the PSRS, the role of the PSRS in health care, and the challenges and decision-making process regarding personalization. We also asked about the role of the PSRS in supporting the health care professional and the health care user in personalization.
2. Health care users: We conducted seven semistructured interviews (average 45 min) with healthy individuals (all adult volunteers) who were recruited for the user study to collect user experiences and expectations regarding the use of the PSRS. Personalization was a theme in the interview. The interviewees were asked to consider how the PSRS could ideally be integrated with their needs, what value this could create, and what type of personalization they would expect to receive while using the PSRS. As the interviewees did not have prior experience with the PSRS or with any other form of human movement analysis system, they each underwent a Posture Scan (at the Swedish research institute) and had a discussion with the health care professional before the interview.
3. Health care professionals: We conducted two group interviews and nine semistructured interviews (average length: 45 minutes) with 13 health care professionals (5 general physiotherapists, 2 personal trainers, 3 occupational physiotherapists, 1 movement therapist, and 2 manual therapists) who worked with health services in physiotherapy. Interview themes concerned the role and use of information systems as part of health service delivery and the professionals’ work practices in general, with more focused questions about the support that the PSRS can provide for the health care professional to treat the individual health care user. As only one health care professional had prior experience with the PSRS, we demonstrated the Posture Scan and reporting procedure of the Movement Lab with paper prototypes before the interviews.

### Data Analysis

The collected data were analyzed thematically [38]. In the thematic analysis, we first familiarized ourselves with the data (a total of 117 A4 pages of transcript material). Second, we analyzed the viewpoints of the different stakeholders separately to form a general understanding of the themes in each viewpoint. Third, we compared the themes between the viewpoints, and the authors held extensive discussions to generate the higher-level themes that we found to be shared by the different stakeholders. As a result, we identified three main types of support that posture scanning can provide to enhance and enable more personalized delivery of physiotherapy.

## Results

**Figure 2:**
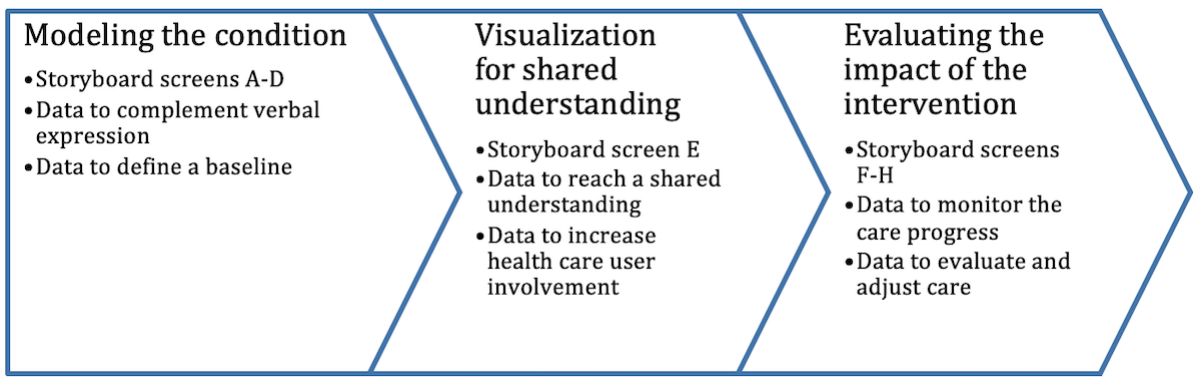
Three types of support posture scanning can provide for data-driven personalization of physiotherapy.

Based on our data analysis, we identified three different types of support that posture scanning can provide to enhance and enable a more personalized delivery of physiotherapy: 1) Modeling the condition, in which the posture scanning data are used to understand the health care user’s current condition and the root cause of the possible problem or pain; 2) visualization for a shared understanding, which includes themes about the use of posture scanning data to enable health care users to be more informed about and involved in their care decisions; and 3) evaluating the impact of intervention, which includes themes related to the use of posture scanning data as a new means for the stakeholders to monitor and evaluate the impact of the intervention. An overview of the three types of support is shown in Figure 2.

### Modeling the Condition

The first type of support concerns the use of posture scanning data to model the current movement levels and condition of the individual health care user who is seeking help. As the health care user consults the health care professional (Screen A in the storyboard) and verbally expresses discomfort, the posture scanning data can provide information to complement the health care user’s verbal description. However, the actual root cause of the problem may be a dysfunction that is challenging for the health care user to verbally describe. With vague descriptions, the evaluation of the health care user’s current condition may take longer; however, the data can support the health care professional in understanding the condition and the possible root cause of the pain (Screen B):

(If the health care user has pain in the knee) the health care professional can see that the knee is not the problem. The problem is the weak hip that causes the pain to the knee.

Information System Developer 1

If the problem of the health care user is not clear [for example difficulty with getting out of the chair], it can be a trigger to do the Posture Scan because that can yield results sometimes. If it is a medical thing you need a physiotherapist, but with vague complaints the Posture Scan can give some useful information.

Health Care Professional 4

The posture scanning data is processed through machine learning algorithms (Screen C) that quantify movement and enable monitoring of progress and potentially identify the cause of pain. The quantification of movement levels supports the health care professional in defining the baseline for care (ie, the care starting point for the individual health care user) (Screen D).

### Visualization for Shared Understanding

The second type of support concerns the use of posture scanning data in reaching a shared understanding on the care options. Posture scanning can be used to visualize the health care user’s movement levels through data displays, which can then be used to guide the discussion between the health care professional and the health care user. The health care users were expecting the health care professional to be active in explaining the care options and leading the decision-making regarding the care options; however, the visualization through data displays also provided a means for the health care user to be more informed and involved in the discussions:

We call this like a communication tool between health care professional and health care user. With this system, the health care professional can say to a health care user that you still have pain, but your movement is much better so keep going with [the rehabilitation].

Information System Developer 1

The communication with the health care user now goes via WhatsApp and that is just easily accessible and quick. Via PC it is a step back. I would use the scan to show progress. Then I can numerically show people that they are doing better.

Health Care Professional 10

The importance of reaching common ground and keeping the health care user informed was also prevalent in the case of motivation. The level of motivation to commit to the exercises was discussed by the health care professionals, as they reported using different motivational techniques and pep talks to address the importance of the exercises. The need for these techniques was apparent, as the health care users were not always motivated to commit to and follow the care options that were verbally discussed and agreed upon at the appointment:

Most people just do not want to do exercises. Of course, these exercises are somewhat boring, but that is why you have to try and find a way and think with your health care user. What they have to do and when.

Health Care Professional 1

Visualization through data displays can be an informative and illustrative way to represent the movement levels to the health care user (Screen E). Visualizing the potentially decreased movement levels can be a more concrete way to illustrate the connection between the potential problem or pain and the care options and prescribed exercises.

### Evaluating the Impact of the Intervention

The third type of support concerns the impact evaluation of the intervention. As the health care user conducts the prescribed exercises using the Movement Lab (Screen F), the aggregated data help the health care professional monitor the care process. The data also provide evidence that the prescribed exercises have been effective (Screen G) and that the movement has potentially improved. In some cases, the health care user’s pain level may not yet have changed, even though their movement levels may have already improved:

If, for example, my back pain has not improved, I expect some personalized help from the health care professional. Maybe exercises need to be changed or the time plan.

Health Care User 1

In such cases, the aggregated data were seen to be especially useful:

I would use the scan to show progress. Then I can numerically show people that they are doing better.

Health Care Professional 10

The evaluation of the impact of the intervention also included a comparison of the aggregated data sets. Comparison of data can show whether the health care user’s movement levels have improved (Screen H). In addition, the data aggregated by Posture Scan and the data that health care users report using Movement Lab can be used as a basis to collaboratively evaluate why the movement levels and pain levels have not (yet) improved:

You can compare scan 1 and 2. The health care user can say that s/he has done the exercises but has not improved. The exercise may be wrong, or the health care user has done the exercise wrongly or has not done the exercises at all.

Information System Developer 2

In the case that their condition did not improve, the health care users expected personalization of care in a way that considered the aggregated data:

If I get exercises to do every day, I will do it, but I also want to do another scan to see the progress. If there has been progress, fine, but if not then I want to contact the health care professional and get an appointment.

Health Care User 5

The aggregated data provide a means for the health care professional and the health care user to consider and evaluate the potentially changed movement levels and pain levels of the health care user. The aggregated data also provide evidence for the health care professional to evaluate the impact of the intervention and to consider with the health care user what to change and do differently; this can result in a more personalized care process.

## Discussion

### Principal Findings

This study investigated how the availability of a new set of digital data, namely posture scanning data, can support and enable more personalized delivery of physiotherapy. We propose that posture scanning data can provide three different types of support to enhance traditional expert-driven physiotherapy and to help personalize the delivery of physiotherapy. The three types of support that we identified and described are 1) modeling the condition, 2) visualization for shared understanding, and 3) evaluating the impact of the intervention. Through our case study, we also described the potential to create value from the data. This knowledge can help to ease the adoption of information systems in physiotherapy, which still remains low in practice [21].

The findings of this study show that posture scanning data can provide new means to deliver and co-create a care service. The aggregated posture scanning data can be used to quantify the health care user’s movement levels, which provides new means for the health care professional to evaluate the health care user’s movement condition. The visualization of data can also be illustrative for the health care user to be more involved and informed regarding their movement and physical therapy. This provides new possibilities to increase the agency of the health care user, which can improve their quality of health (eg, through better adherence) [39].

Our findings show how the posture scanning data can be blended into the traditional physiotherapy service encounter and how they can be seamlessly integrated into the care practice. Increasing amounts of data about the individual health care user can be aggregated through different devices and sensors. The findings of this study increase our understanding of the value of these data for the core stakeholders in the context of physiotherapy.

### Comparison With Prior Work

As people collect more personal data about themselves and organizations store information on their interactions with people [40], data are becoming a core component of current care models [12]. Information systems are among the main facilitators of these care models; they facilitate the integration of different data sets and best care practices to enable seamless health service delivery in a person-centered manner [3,4,11]. The aggregated data can support health care professionals in complementing the health care user’s own resources in managing their health [6]. As health care users are also increasingly using different technologies and data sources to manage their health [41], health care professionals may need to be ready to support health care users in interpreting and using the data stored and processed by the various different technologies used in care [42-44]. This study contributes to this area of research and practice by providing insights on how the availability of digital data can enhance and personalize physiotherapy services. The findings are derived from the insights gathered from the stakeholders. This is rarely done in the field of personalization [45]. In their study, Cena et al [45] used a personal informatics system to collect evidence from users to aid the research and practice of personalized services. The findings of this study therefore extend this area of personalization research, where personalization is examined through the viewpoints of stakeholders who co-created the service.

Lee [30] proposed a model of health care service pathway design which takes into account the influence of different service encounters by technologies (including mobile apps and websites), health care user interactions with health care professionals, and care service outcomes on the health care user experience. Our study provides a concrete example of how advanced technology in the form of a posture scanning system can be integrated into the design of physiotherapy services and of the different types of support such a system can provide for the interaction between the physiotherapist and health care user; this will hopefully result in better health service outcomes.

Service design is not often used in health care [46], as different quantitative approaches and statistical outcomes have been more valued than qualitative methods [47]. The service design approach considers the needs and expectations of the different stakeholders who are involved in and impacted by the service [27]. More recently, the service design approach has shown benefits in the design and implementation of different health care technologies, such as the implementation of an electronic health record (EHR) [28]. The continuous development of different technologies provides new possibilities for the design of service encounters [48]. This study demonstrates the use of the service design approach to explore the needs and expectations of different stakeholders who are involved in and impacted by the service. Our study shows how these viewpoints can be taken into account in the design of information system solutions and new care models that can better support personalization.

### Limitations

Like most research, this study has limitations. First, the reported case study was conducted in Northern European countries, and all the study participants were working in these countries. Therefore, this study does not account for cultural differences worldwide, which can be considered as a limitation to the generalizability of the results. Second, we collected data from different stakeholder viewpoints; however, for ethical reasons, the health care users were healthy individuals who were recruited through purposeful sampling. However, as the PSRS can also be used to provide data for healthy individuals who are interested in their movement levels, we believe that even though the users were not currently in need of physiotherapy, they were capable of reliably expressing a user’s viewpoint, opportunities, and expectations regarding personalization.

### Conclusions

Health care is becoming increasingly data-driven. The availability of digital data can be used for data-driven personalization, providing opportunities to align the care process more closely with the needs of an individual health care user in a person-centered manner. In physiotherapy, the adoption of digital tools that can support rehabilitation practice has remained low; however, personalization is considered to be the key factor in the adoption of digital technologies. This study contributes to this area of research by proposing three different types of support that digital data acquired from posture scanning can provide to enhance and enable more personalized delivery of physiotherapy. The results of this work provide insights for the design of personalized health care services that consider the viewpoints of different stakeholders in the future of personalization. Future research could also investigate and measure the effects of using a posture scan system and its data on the outcome of the physiotherapy service—for example, does the use of the system shorten the time required for health care users to recover from a certain type of health problem or overcome pain in a specific body part? Future research could also investigate in more detail how the viewpoints of the three different stakeholder groups—health care professionals, health care users, and information system providers—should be integrated when designing personalized health care services.

## Abbreviations

AI: artificial intelligence
EHR: electronic health record
ICD-10: International Classification of Disease, Tenth Revision
PSRS: posture scan recording system
